# Small bowel intussusception due to adenocarcinoma of ectopic pancreas in the jejunum: a case report

**DOI:** 10.1186/s40792-024-02060-z

**Published:** 2024-11-19

**Authors:** Kota Yamamoto, Takahiro Ishimori, Taiki Okada, Takeshi Sasaki, Yumi Mikajiri, Takahiro Terashima, Shunji Kawamoto

**Affiliations:** Department of surgery, Shonan Atsugi Hospital, Nurumizu 118-1, Atsugi, Kanagawa 243-0033 Japan

**Keywords:** Adenocarcinoma of ectopic pancreas in the jejunum, Small bowel intussusception, Adenocarcinoma

## Abstract

**Introduction:**

We encountered a case of adenocarcinoma of the ectopic pancreas, causing intussusception.

**Case presentation:**

A 76-year-old man presented with complaints of abdominal distention and vomiting to the emergency department in March 2022. Computed tomography showed that the small bowel piled up approximately 20 cm from the ligament of the traits. Endoscopic repair was challenging; therefore, laparoscopic repair and partial resection of the small bowel were performed. The specimen showed a mass in the small bowel arising from an ectopic pancreas that had caused accumulation. Pathological examination revealed ectopic pancreatic cancer. Two years postoperatively, no apparent recurrence has been observed. We report a relatively rare case of a cancerous ectopic jejunal pancreas causing a mass, with a discussion in the literature.

**Conclusions:**

Detection typically requires surgery due to advanced-stage intestinal obstruction or accumulation, as observed in the present case. However, preoperative diagnosis and early detection of ectopic pancreatic cancer are challenging. The disease progresses similarly to pancreatic cancer, highlighting the need for early detection methods. Additionally, accumulating more case reports is essential for establishing an effective treatment strategy.

## Introduction

Here, we report a case of adenocarcinoma of the ectopic pancreas that caused intussusception.

Reports of carcinomatosis of ectopic pancreatic cancer are rare [1], and the only reported cases discovered as a result of bowel accumulation were our self-examined cases.

## Case presentation

**Fig. 1:**
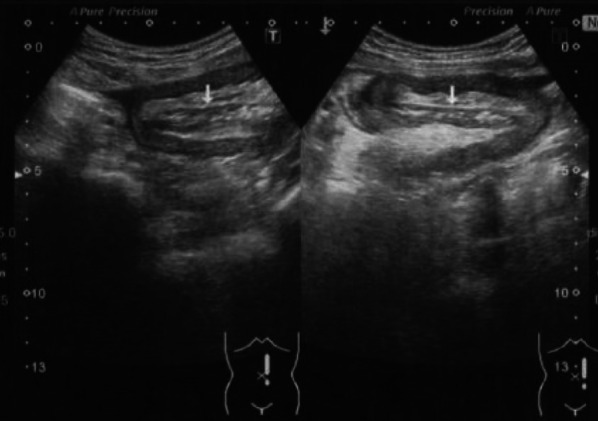
Abdominal ultrasonography. A small amount of ascites in the cysto-rectal fossa, a high echogenic mesentery, and small intestinal insertions in the intestinal tract of the left abdomen can be noted. Oral bowel dilatation was also observed

**Fig. 2:**
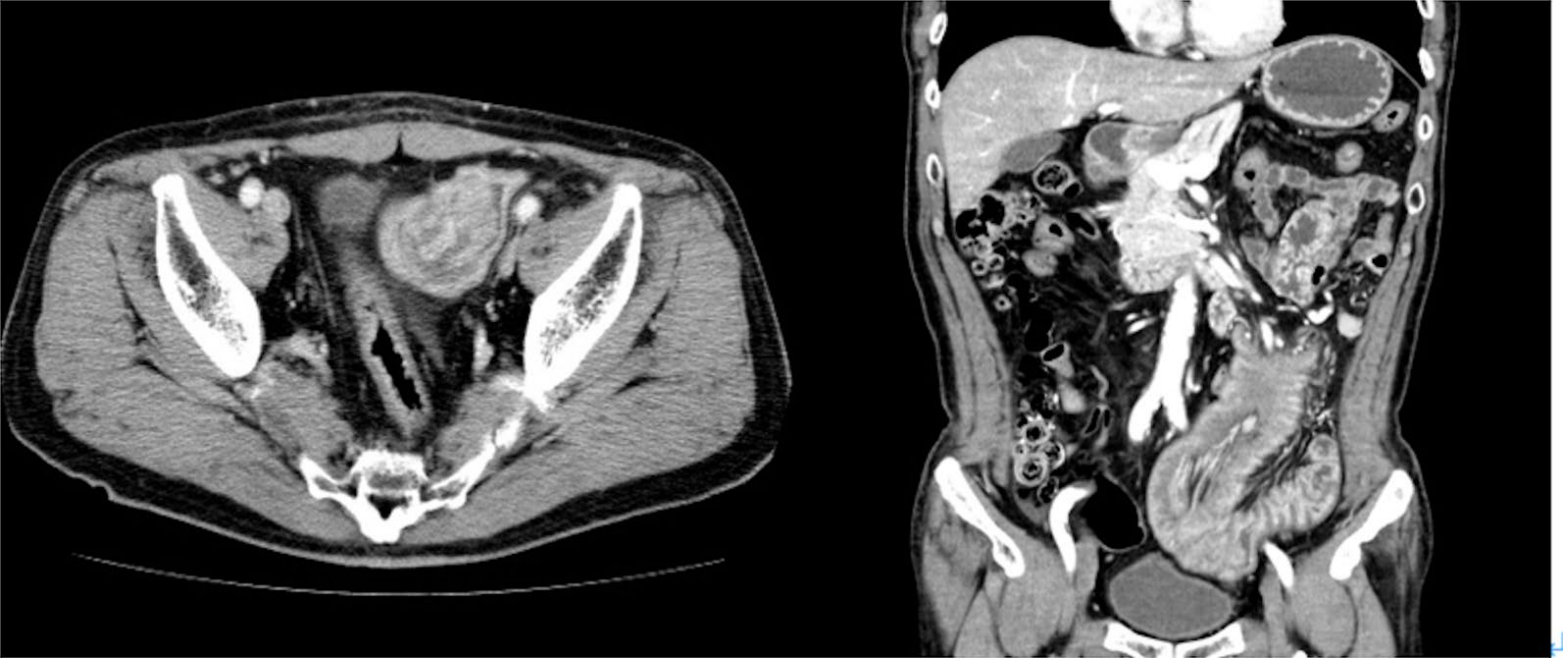
Abdominal contrast-enhanced computed tomography. The image shows a 20-cm-long small bowel–small bowel type superimposed findings with a preceding mass with sparse contrast in the jejunum. No apparent pancreatic abnormalities were observed

A 76-year-old man visited another hospital with chief complaints of abdominal pain and distention. The patient had no specific medical or family history. On examination, a mass was palpable in the upper abdomen. He was referred to our hospital for surgical treatment after a thorough examination using abdominal X-ray and ultrasonography (Fig. 1). Simple computed tomography (CT) revealed an intussusception in the jejunum. Therefore, we performed a contrast-enhanced CT scan (Fig. 2), which showed a preserved contrast effect on the bowel wall. Staging laparoscopy was performed since non-obstructive repair via gastrointestinal endoscopy was difficult.

**Fig. 3:**
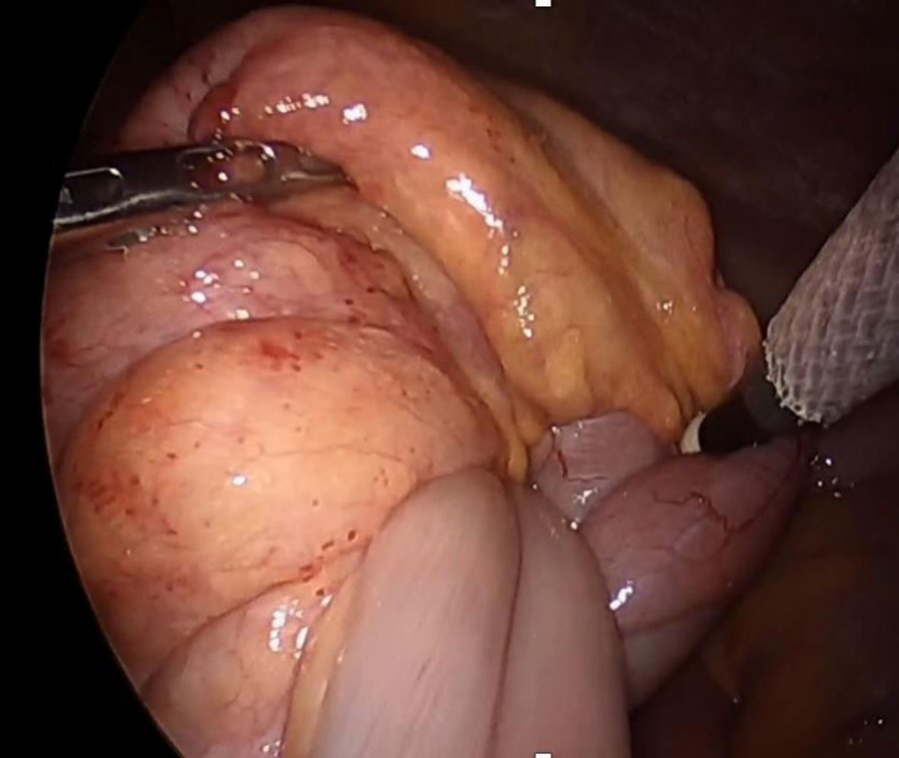
Intraoperative findings. The accumulation was easily released by the Hutchinson maneuver using laparoscopic forceps. No ischemic necrosis was noted in the area of the small bowel where the accumulation had occurred

A small bowel-type accumulation was observed approximately 10–30 cm from the ligament of Treitz (Fig. 3). The accumulation was easily released by the Hutchinson maneuver using laparoscopic forceps. No ischemic necrosis was found in the area of the small bowel where the accumulation had occurred. The lesion was removed from the body, and an elastic hard mass was identified on palpation of the bowel. Partial small bowel resection was performed with a 10 cm margin before and after the mass. Next, the specimen was opened to confirm the presence of the mass, which was a yellow elevated submucosal tumor 3 cm in size with a relatively firm, uneven, and irregular mucosal lesion suspected to be small bowel cancer. The surgery was completed with additional dissection of the small bowel mesentery in the D2 resected area.

**Fig. 4:**
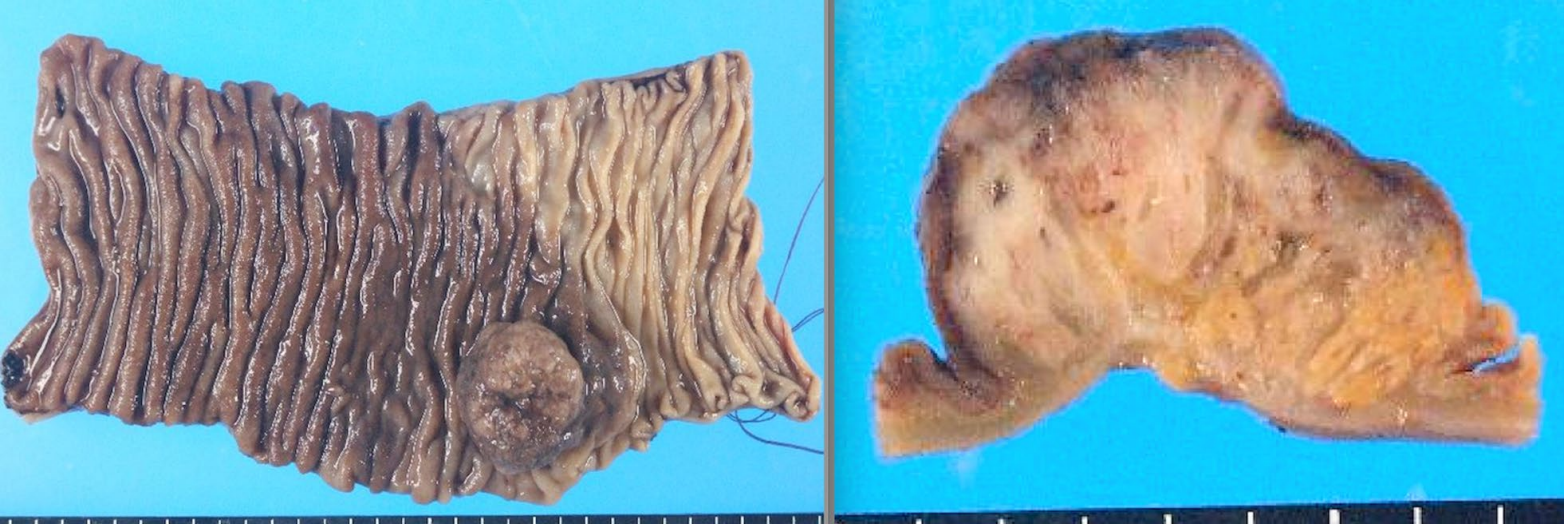
Macroscopic findings. The lesion was diagnosed as invasive ductal carcinoma, papillary adenocarcinoma, arising from the ectopic pancreas of the jejunum. The specifics included INFα, depth mp, ly0, v1, n0, with a tumor diameter of 20 × 20 mm

**Fig. 5:**
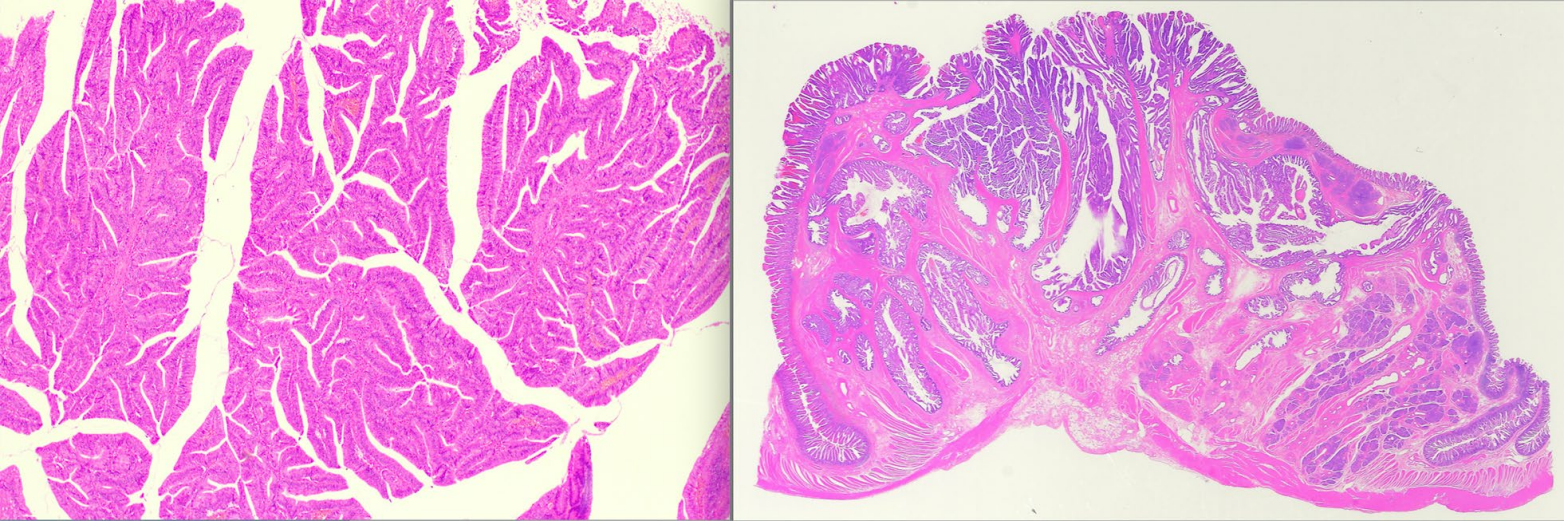
Pathological findings. Pathological diagnosis revealed pancreatic ducts and acinar cells with no islets of Langerhans, indicating Heinrich classification type II. Tumor cells predominantly proliferated from the submucosa of the jejunum to the intrinsic muscularis mucosae. Simultaneously, an abrupt transition (conflict between normal and neoplastic epithelia) of the normal glandular ducts and malignant findings on the mucosal surface was noted

Pathological diagnosis (Figs. 4, 5) revealed pancreatic ducts and acinar cells with no islets of Langerhans, indicating Heinrich classification type II [2]. Tumor cells predominantly proliferated from the submucosa of the jejunum to the intrinsic muscularis mucosae. Simultaneously, an abrupt transition (conflict between normal and neoplastic epithelia) of the normal glandular ducts and malignant findings on the mucosal surface was noted. Originally, no findings in the pancreas were suggestive of the primary tumor.

The ectopic pancreatic cancer was classified as pT2N0M0 stage I according to the Union for International Cancer Control-Tumur, Node, Metastasis (UICC-TNM classification, 8th edition, 2017) [3]. According to the National Cancer Center and Rare Cancer Center website [4], resection of the lesion is the primary treatment, and we successfully resected it laparoscopically. Postoperative adjuvant chemotherapy for adenocarcinoma of the small intestine is currently undergoing phase III clinical trials. According to the treatment guidelines for pancreatic cancer, the patient underwent R0 surgery at stage I although postoperative adjuvant chemotherapy was necessary. We recommended S-1 as a first choice; however, he chose not to pursue it. Fortunately, the patient has survived without a recurrence for 2 years.

## Discussion

The most common cause of ectopic pancreas is generally believed to be stray pancreatic tissue during fetal development. In addition, the most common sites of ectopic pancreas are the duodenum and stomach, which are close to the pancreas, followed by the jejunum, ileum, and Meckel’s diverticulum [5]. The condition predominantly affects males, with a male-to-female ratio of 2:1. Typically, ectopic pancreas does not present with clinical symptoms. Moreover, Heinrich classification [2] is a well-known tissue classification method. Type I has islets of Langerhans, adenocytes, and conduits and has the same structure as normal pancreatic tissue; type II lacks islets of Langerhans and comprises adenocytes and conduits; type III lacks islets of Langerhans and adenocytes and comprises only conduits; and type IV comprises only islets of Langerhans without pancreatic exocrine tissue. Based on pathological findings, our case was classified as Heinrich type II.

Although multiple reports of ectopic pancreatic carcinogenesis exist in the past, our review of the Non-Profit Organization (NPO) Japan Medical Abstracts Society database from 2000 to 2023 under the terms “jejunum” and “ectopic pancreatic cancer” revealed 22 reports, including our cases. However, the only reported cases discovered as a result of bowel accumulation were our self-examined cases (excluding conference proceedings).

The criteria for the transformation of the ectopic pancreas into carcinoma, according to Mibayashi et al. [6], are as follows: (1) the lesion must be observed grossly, mainly from the submucosa to the muscularis mucosae, and show no association with the mucosa except for mucosal perforation; (2) the ectopic pancreas must be complicated or coexist, and the tumor must show evidence of a transition from these lesions to carcinoma or the histological picture of the tumor must resemble that of pancreatic cancer; and (3) the tumor must not have metastasized from pancreatic cancer. In this case, the patient was found to have intestinal accumulation, and the intraoperative specimen was opened due to suspicion of jejunal carcinoma exposed on the mucosa. Therefore, the lymph nodes of the patient were dissected.

In this case, the pathological findings showed a Heinrich type II ectopic pancreas and a tumorigenic image of the pancreatic duct, mainly in the submucosal layer, extending from the abrupt transition part of the mucosal surface to the intrinsic muscular layer. As the contrast-enhanced CT scan showed a normal pancreas, we considered that the case met the definition of ectopic pancreatic cancer of the jejunum. The size was 3.5 cm, depth was in the intrinsic muscular layer, and no lymph node metastases were observed. According to previous literature, 80% of lesions are 1–3 cm in size, and most of them are located from the submucosa to the muscularis propria [7]. When diagnosed with ectopic pancreatic cancer, Hirata et al. [8] recommended treatment according to pancreatic cancer based on histological features. The need for lymph node dissection, in addition to lesion resection, has been highlighted.

According to the National Cancer Center and Rare Cancer Center website [4], small intestine cancer generally refers to duodenal, jejunal, and ileal cancers, with neuroendocrine tumors, adenocarcinomas, malignant lymphomas, and sarcomas (gastrointestinal stromal tumors and leiomyosarcoma) as the most common histologic types. It is considered a rare type of cancer, accounting for < 0.5% of all malignant tumors and < 5% of all malignant gastrointestinal tumors. Small intestinal adenocarcinoma is staged according to the UICC-TNM classification, although its treatment has not yet been established. While resection of the lesion is a curative treatment, the need for dissection and the resecting area is yet to be clearly elucidated. Moreover, the effectiveness of adjuvant chemotherapy after radical surgery has not been established, and no drugs are covered by insurance. The efficacy of fluoropyrimidine + oxaliplatin therapy has been demonstrated in stage IV patients with distant metastases or recurrent disease, and fluorouracil, leucovorin, and oxaliplatin (FOLFOX) is also covered by insurance in Japan. However, only approximately 10% of a relatively high rate of MSI-positive patients are known, and the efficacy of pembrolizumab has been demonstrated.

Hoshino et al. [1] reported 20 cases of ectopic pancreatic cancer of the jejunum. A total of 22 cases exist, including our case. Among these, eight cases had lymph node metastasis or distant metastasis at surgery, and eight experienced recurrence within 1 year after radical resection, all with a poor prognosis. All patients were treated with chemotherapy according to the guidelines for pancreatic cancer treatment. In addition to the study by Miyazaki et al. [9], eight cases of so-called N0 and M0 have been reported, as in the present case, although the exact staging was difficult to determine as the depth of some cases was unknown. Moreover, the optimal duration of chemotherapy administration is unknown. Table 1 presents the postoperative recurrence and survival of these seven patients with early-stage cancer, with or without adjuvant chemotherapy. Our patient did not receive postoperative adjuvant chemotherapy but survived 24 months postoperatively without recurrence.

**Table 1:** Past reported cases of adenocarcinoma of ectopic pancreas in the jejunum

Case	Authors	Year	Age	Sex	Heinrich type	Metastasis	Adjuvant	Postoperative course
1	Fujiki [12]	1990	54	M	I	Liver	MTX/5-FU	Death in 7 months
2	Sato [13]	1993	64	M	I	Lymph node	Unknown	Recurrence peritoneal in 3 months
3	Saegusa [14]	1995	76	F	I	None	Unknown	No recurrence for 14 months
4	Arao [15]	1999	63	M	II	Liver Lymph node	Unknown	Unknown
5	Uemura [16]	2001	72	M	II	Lymph node	Unknown	No recurrence in 6 months
6	Matsubara [17]	2004	79	M	Unknown	None	None	Peritoneal recurrence in 6 months and death in 9 months
7	Sato [18]	2007	78	F	II	Unknown	Unknown	Unknown
8	Koh [19]	2009	50	M	Unknown	Peritoneum Greater omentum	None	Peritoneal recurrence in 2 weeks and death in 6 weeks
9	Ono [20]	2010	51	F	III	Unknown	UFT/GEM	Peritoneal recurrence in 7 months and death in 28 months
10	Nagai [21]	2014	70	M	II	Peritoneum	GEM	Death in 4 months
11	Okada [22]	2014	71	M	I	None	GEM/S-1	Liver recurrence in 10 months
12	Kurata [23]	2014	70	M	I	Unknown	Unknown	Unknown
13	Ogawa [24]	2015	70	M	I	None	GEM/S-1	No recurrence in 22 months
14	Matsuyama [25]	2015	72	F	II	Peritoneum	None	Death in 1 month
15	Ito [26]	2016	66	F	II	None	S-1	No recurrence in 3 months
16	Miyazaki [9]	2016	72	M	I	None	S-1	No recurrence in 24 months
17	Yogi [27]	2017	82	M	I	Unknown	S-1	No recurrence in 7 months
18	Sakoda [28]	2018	91	M	Unknown	None	None	Death in 4 months
19	Shigemasa [29]	2019	96	F	II	Unknown	None	Death in 6 months
20	Sakurai [30]	2019	69	F	unknown	Peritoneum	GnP	No recurrence in 6 months
21	Hosino [1]	2022	82	M	II	None	None	Liver recurrence in 2 months
22	Our case	2023	76	M	II	None	None	No recurrence in 24 months

M, male; F, female; GEM, gemcitabine; GnP, gemcitabine and nab-paclitaxel; MTX/5-FU, methotrexate/5-fluorouracil

S-1 and gemcitabine (GEM) are recommended as adjuvants for pancreatic cancer [10, 11]. As mentioned above, no clear evidence of small intestinal cancer exists. If jejunal ectopic pancreatic cancer is treated as pancreatic cancer, S-1 or GEM would be the treatment of choice. Miyazaki et al. [9] suggested the efficacy of adjuvant S-1 as well as pancreatic cancer. In our case, adjuvant therapy was indicated as the treatment of choice because the pathological findings revealed a tumor with vascular invasion. Although we recommended S-1 as a first choice, we decided to follow up without adjuvant therapy as per the patient’s choice. Fortunately, the disease has not recurred, although we are still carefully monitoring the patient’s progress, partly due to the abovementioned reasons.

In the case of advanced pancreatic cancer, other options are available in accordance with the guidelines for pancreatic cancer treatment; however, the prognosis is extremely poor, even in the cases reported to date. Therefore, as with pancreatic cancer, early detection of ectopic pancreatic cancer is required; however, similar to tumors of the small intestine, it takes time for symptoms to appear. Previously, many patients visited hospitals with abdominal pain and nausea, and intestinal obstruction was found upon close examination. In our case, the patient had a stacked intestine, although the cause of vomiting associated with dilatation of the oral intestinal tract was the same.

## Conclusions

For detection, the patient must undergo surgery owing to intestinal obstruction at an advanced stage or intestinal accumulation, as observed in the present case, and because distinguishing ectopic pancreatic cancer in the preoperative diagnosis or early detection is challenging. The disease progresses similarly to pancreatic cancer, making early detection methods crucial. Therefore, accumulating more case reports in the future is necessary to establish an effective treatment strategy.

## Data Availability

Deidentified patient data will be available upon reasonable request to the corresponding author.

## Abbreviations

CT: Computed tomography
UICC-TNM: Union for International Cancer Control—tumor, lymph nodes, metastasis
NPO: Non-profit organization
FOLFOX: Fluorouracil, leucovorin, oxaliplatin
GEM: Gemcitabine
